# Effects of ensiling period and lactic acid bacteria inoculation on aerobic stability of silage in forage infected with leaf spot disease

**DOI:** 10.3389/fmicb.2025.1548678

**Published:** 2025-04-28

**Authors:** Guojian Tang, Jinmei Yang, Yuanyan Meng, Xiaolong Zhang, Mengxin Wen, Ting Sun, Dan Wu, Liuxing Xu

**Affiliations:** ^1^School of Biological Sciences and Technology, Liupanshui Normal University, Liupanshui, China; ^2^College of Agronomy and Life Sciences, Zhaotong University, Zhaotong, China

**Keywords:** aerobic exposure, leaf spot disease, silage fermentation quality, aerobic stability, ensiling period, additives

## Abstract

When forage crops are ravaged by leaf spot disease, producers face an unavoidable dilemma: a careful consideration of adopting specific measures to make use of the damaged forage. Furthermore, silage is often exposed to air during production, feeding, storage, and transportation. The aim of this study is to investigate the effects of three ensiling periods (15, 30, and 60 days), two crops (Italian ryegrass, and oats), and three additives [control group (CK), inoculated with *Lactobacillus plantarum* YM3, and *L. rhamnosus* HT1] on the health and fermentation quality of silage infected with leaf spot disease during aerobic exposure. Silage ensiled for 15 days had the highest lactic acid concentration and the lowest butyric acid concentration (*p* < 0.05). The acetic acid concentration of Italian ryegrass silage (5.77 g kg^−1^ DM) was higher than that of oat silage (2.89 g kg^−1^ DM), and the butyric acid concentration was lower (2.70 g kg^−1^ DM versus 5.94 g kg^−1^ DM) (*p* < 0.05). The lactic acid concentration of silage inoculated with *L. rhamnosus* HT1 (92.0 g kg^−1^ DM) was significantly higher than that inoculated with *L. plantarum* YM3 (57.3 g kg^−1^ DM) and the CK (69.5 g kg^−1^ DM) (*p* < 0.05). For most undesirable bacterial species, such as *Stenotrophomonas*, *Providencia*, *Paenalcaligenes*, *Myroides*, and *Alcaligenes*, the relative abundances in the silage ensiled for 60 d were generally higher than in those ensiled for 15 and 30 days. The relative abundance of harmful bacteria in oat silage was higher than that in Italian ryegrass silage. The relative abundances of *Stenotrophomonas* and *Providencia* in the CK treatment were higher than those in the silage inoculated with *L. plantarum* YM3 and *L. rhamnosus* HT1. The addition of lactic acid bacteria (LAB) helped to inhibit the increase in the relative abundance of harmful bacteria. Therefore, when silage has to be exposed to air, this study recommends that Italian ryegrass be inoculated with *Lactobacillus rhamnosus* HT1 and used within 15 days of ensiling.

## Introduction

Silage, a crucial feed source in animal husbandry, directly influences the growth rate and health of livestock (Little et al., 2017). However, forage crops often encounter various disease challenges during their growth, with leaf spot disease being the most prevalent (Victoria Arellano et al., 2022). To date, over 40 countries have reported cases of leaf spot disease, primarily affecting alfalfa (Lan et al., 2024). This disease not only causes yield losses ranging from 31% to 82% in greenhouse environments and 56% in field conditions but also severely compromises the nutritional composition of forage, potentially impacting livestock reproductive capability, particularly posing a significant risk to ovulation rates (Lan et al., 2024). However, when forage crops are ravaged by leaf spot disease, producers face an unavoidable dilemma. They must carefully consider adopting specific measures to make use of this damaged forage, as seeking alternative feed sources often entails a greater economic burden. Therefore, effectively enhancing the quality of silage made from forage infected with leaf spot disease has emerged as a core challenge that needs to be addressed in the animal husbandry sector. Lactic acid bacteria (LAB) are probiotics that play a pivotal role in silage fermentation (Okoye et al., 2023). Through their unique fermentation mechanism, they significantly reduce the pH of forage, effectively inhibiting the growth of harmful microorganisms, thereby extending the shelf life of silage and improving its fermentation quality (Jiang et al., 2020). However, the current understanding of how LAB additives regulate fermentation quality and microbial community health of silage made from forage infected with leaf spot disease remains unclear.

Silage duration is a key factor that affects silage quality. If the silage period is too short, the feed may not ferment sufficiently; conversely, a period that is too long may lead to significant nutrient loss. Although prolonged silage duration can inhibit the growth of harmful microorganisms, it often results in greater nutrient loss. For example, when the silage duration increases from 0 to 45 days, the ammonia nitrogen content in whole-plant maize (*Zea mays*) silage increases from 15.5–20.0 (g kg^−1^ total N) to 31.9–41.7 (g kg^−1^ total N) (Huang et al., 2021), and this situation may be exacerbated in alfalfa (*Medicago sativa*) (Gao et al., 2021). However, after the fermentation period exceeds 7 days, LAB in silage are primarily beneficial microorganisms such as *Weissella* and *Lactobacillus* (Li et al., 2020). Therefore, selecting an appropriate duration is crucial for improving the fermentation quality of silage. Additionally, silage is often exposed to air during production, feeding, storage, and transportation (Bolsen, 2018; Yin et al., 2021). Aerobic exposure increases the oxygen content of silage, promoting the growth and reproduction of aerobic microorganisms, thereby affecting its fermentation quality and microbial community structure (Zhang et al., 2023). Therefore, exploring how aerobic exposure and silage duration affect the fermentation quality and microbial health status of silage is crucial for improving storage and transportation conditions, enhancing nutritional value, and extending shelf life. However, current research in this field, particularly in-depth studies on silage fermentation quality and microbial health, remains relatively scarce, and the details and underlying mechanisms require comprehensive elucidation.

This study focused on exploring the variation patterns in microbial community health and silage fermentation quality of forage infected with leaf spot disease after treatment with LAB and subjected to different ensiling periods under aerobic conditions. Based on this, the study proposes the following two hypotheses: (1) the extension of silage duration may promote the improvement of microbial health in silage exposed to air, and (2) the addition of LAB may lead to an improvement in the health status of silage exposed to air.

## Materials and methods

### Materials and silage additives

Italian ryegrass and oats were cultivated in the experimental field (27°33′N, 103°77′E) at Zhaotong University (Zhaotong, China). Forage with > 30% leaf spot was selected for ensiling when the Italian ryegrass and oats were at the flowering and milking stages, respectively. These materials were chopped into 20–30 mm for silage preparation. The silage additives used were *L. plantarum* YM3 (YM3) and *L. rhamnosus* HT1 (HT1), and the strains were obtained from the forage processing laboratory of South China Agricultural University.

### Experimental design and silage preparation

The experimental design included three ensiling periods (15 days, AS15; 30 days, AS30; 60 days, AS60), two crop types (Italian ryegrass, IR_A; oats, Oat_A), and three additives (without, ASCK, YM3, ASYM3; HT1, and ASHT1). The chopped materials were mixed thoroughly with the three additives separately, packed into polyethylene plastic bags (200 × 300 mm; Zhongshan, China), and sealed with a vacuum sealer (P-290; Shenzhen, China). Strains YM3 and HT1 were inoculated at a rate of 1.0 × 10^5^ colony-forming units (cfu)/g fresh matter (FM), and appropriate amounts were added to the sterilized small spray and diluted with 10 mL of sterile water. An equal volume of sterile water was added to the chopped material in the control group (CK). Strains YM3 and HT1 require activation in de Man, Rogosa, and Sharpe (MRS) broth medium before inoculation and detection of viable bacteria by the plate count method (Chen et al., 2022). The plastic bag silos were stored in a laboratory cabinet (15°C–25°C, dark environment) for 15, 30, and 60 days, respectively. The plastic bag silos were opened at the designated ensiling time and aerobically exposed for 7 days at room temperature (15°C–25°C) to analyze the microbial, fermentation and chemical characteristics.

### Microbial population and fermentation quality analyses

The number of microorganisms was determined using a culture medium cultivation method (Chen X. et al., 2021; Tang et al., 2023). LAB, aerobic bacteria, yeasts, and molds were counted on MRS, nutrient, and potato dextrose agar, respectively. LAB were cultured under anaerobic conditions at 37°C for 2 days. Aerobic bacteria, yeasts and molds were cultured under aerobic conditions at 37°C for 3 days.

The pH of the filtrate (10 g fresh material added to 90 mL distilled water) was determined using a pH meter (LE438; Mettler Toledo, Shanghai, China). After measuring the pH, the filtrate was used to determine the organic acid concentration (using a small amount of cation-exchange resin). The organic acid concentration (lactic, acetic, propionic, and butyric acids) was measured using high-performance liquid chromatography (column: Sodex RS Pak KC-811, Showa Denko K.K., Kawasaki, Japan; detector: diode array detector, 210 nm, serial presence detect-20A, Shimadzu Co., Ltd., Kyoto, Japan; eluent: 3 mmol L^−1^ HClO_4_, 1.0 mL/min; temperature: 60°C) (Li et al., 2016).

### Bacterial community analyses

According to the method described by Wang et al. (2020), a 10 g frozen sample was placed in 40-mL sterile water, and after homogenization, filtered with two layers of sterile medical gauze, subsequently, the gauze was rinsed with 40 mL sterile water three times to recover the residual microbes, which were recycled using a centrifuge at 12,000 g for 15 min at 4°C after the filtrate was combined. Genomic DNA was extracted from the silage samples using a bacterial DNA isolation kit (DE05311; Foregene, Chengdu, China). DNA concentration and purity were detected using NanoDrop2000C, with the optical density set at 260/280 nm (Chen et al., 2022). Qualified DNA samples were used for subsequent analyses. The DNA samples were paired-end sequenced using the Illumina NovaSeq6000 platform. The sequencing data were analyzed using the Majorbio Bio-Pharm cloud platform. The community structure was analyzed at the phylum and genus levels using the Silva database with a confidence threshold of 90% (Wang et al., 2020). Alpha diversity at the operational taxonomic unit (OTU) level was applied to analyze the complexity of species diversity for a sample through Chao and Shannon indices, and principal coordinate analysis was performed to obtain the bacterial community structural variance. Spearman’s correlation heatmap analysis was conducted to explore the relationship between the microbial community and fermentation products.

### Statistical analyses

The plate count results for microorganisms were log-transformed before statistical analysis. One-and multi-way analysis of variance were performed using SPSS (version 25.0) for ensiling time, crop type, and additives. Duncan’s test was used to evaluate differences among treatments. Differences were considered statistically significant at *p* < 0.05. The model used was: Y = *μ* + Tj + εij, where Y was the response variable, μ was the overall mean, Tj was the treatment effect, and εij was the residual error.

## Results

### Effects of ensiling time, crop type and additives on organic acids and microbial number of silage after aerobic exposure

Regarding ensiling time, the number of aerobic bacteria in the AS15 treatment reached its peak compared to that in the AS30 and AS60 treatments (*p* < 0.05). The number of LAB gradually increased with prolonged silage duration and reached its highest value in the AS60 treatment (*p* < 0.05). The numbers of molds and yeasts in the AS30 and AS60 treatments were significantly higher than those in the AS15 treatment (*p* < 0.05). However, the effects of crop type and additives on the number of microorganisms were not significant (Table 1).

**Table 1:** Microbial counts, pH, and organic acids changes of silage after exposed to the air from different additives, crop types, and ensiling time (*n* = 54).

Treatments		Microbial counts (lg cfu g^−1^ FM)				pH	Organic acids (g kg^−1^ DM)			
		Aerobic bacteria	Lactic acid bacteria	Molds	Yeasts		Lactic acid	Acetic acid	Propionic acid	Butyric acid
Ensiling time (T)	AS15	7.88 ± 0.02a	7.19 ± 0.08c	5.66 ± 0.20b	5.26 ± 0.19b	3.93 ± 0.05b	89.3 ± 4.96a	3.34 ± 0.59b	0.74 ± 0.13	2.47 ± 0.62b
	AS30	7.62 ± 0.09b	7.69 ± 0.05b	6.62 ± 0.09a	6.73 ± 0.12a	3.95 ± 0.05b	71.8 ± 4.11b	2.79 ± 0.56b	2.14 ± 1.41	5.74 ± 1.20a
	AS60	7.68 ± 0.08b	7.93 ± 0.05a	6.73 ± 0.10a	6.77 ± 0.07a	5.52 ± 0.29a	59.7 ± 8.27b	6.38 ± 0.99a	0.21 ± 0.06	5.29 ± 1.38a
Crop type (CP)	IR_A	7.72 ± 0.07	7.66 ± 0.06	6.35 ± 0.17	6.19 ± 0.23	4.17 ± 0.06	79.7 ± 4.75	5.77 ± 0.75a	1.78 ± 1.06	2.70 ± 0.69b
	Oat_A	7.74 ± 0.05	7.52 ± 0.10	6.32 ± 0.13	6.30 ± 0.13	4.71 ± 0.24	65.9 ± 5.94	2.89 ± 0.50b	0.43 ± 0.05	5.94 ± 0.99a
Additive (A)	ASCK	7.87 ± 0.03	7.44 ± 0.09	6.30 ± 0.18	6.37 ± 0.18	4.58 ± 0.25	57.3 ± 5.55b	3.05 ± 0.83b	2.41 ± 1.34	5.75 ± 1.43
	ASYM3	7.66 ± 0.08	7.67 ± 0.10	6.13 ± 0.22	6.12 ± 0.28	4.53 ± 0.28	71.3 ± 5.93b	3.50 ± 0.92b	0.39 ± 0.07	4.25 ± 0.93
	ASHT1	7.66 ± 0.09	7.70 ± 0.09	6.57 ± 0.11	6.26 ± 0.18	4.30 ± 0.20	92.0 ± 5.86a	5.95 ± 0.52a	0.29 ± 0.07	3.50 ± 1.01
*p* value	T	0.028	0.000	0.000	0.000	0.000	0.004	0.002	0.234	0.009
	CT	0.842	0.202	0.915	0.669	0.060	0.073	0.002	0.159	0.014
	A	0.078	0.101	0.214	0.719	0.700	0.000	0.023	0.121	0.375
	T × CT	0.000	0.008	0.094	0.000	0.000	0.020	0.000	0.178	0.339
	T × A	0.202	0.100	0.000	0.004	0.866	0.701	0.326	0.264	0.028
	CT × A	0.820	0.723	0.470	0.984	0.845	0.808	0.286	0.135	0.823
	T × CT × A	0.022	0.278	0.129	0.438	0.162	0.517	0.573	0.210	0.002

Different lowercase letters in the same column represent significant difference among ensiling time, crop types or additives (*p* < 0.05). FM, fresh matter; DM, dry matter; AS15, silage ensiled for 15 days; AS30, silage ensiled for 30 days; AS60, silage ensiled for 60 days; IR_A, Italian ryegrass silage; Oat_A, oat silage; ASCK, no additive silage; ASYM3, silage inoculated with *Lactobacillus plantarum* YM3; ASHT1, silage inoculated with *Lactobacillus rhamnosus* HT1.

Regarding silage fermentation quality, the pH of the AS60 treatment was significantly higher than those of the AS15 and AS30 treatments (*p* < 0.05). However, crop type and additives did not significantly affect the pH. The AS15 treatment resulted in the highest lactic acid and lowest butyric acid concentrations (*p* < 0.05) (Table 1). The acetic acid concentration in the IR_A treatment (5.77 g kg^−1^ DM) was significantly higher than that in the Oat_A treatment (2.89 g kg^−1^ DM), and a lower butyric acid concentration was observed in the IR_A treatment (2.70 g kg^−1^ DM versus 5.94 g kg^−1^ DM) (*p* < 0.05). The ASHT1 treatment (92.0 g kg^−1^ DM) had a higher lactic acid concentration than those of the ASYM3 (57.3 g kg^−1^ DM) and ASCK (69.5 g kg^−1^ DM) treatments (*p* < 0.05).

### Effects of ensiling time, crop type and additives on silage bacterial diversity after aerobic exposure

**Figure 1:**
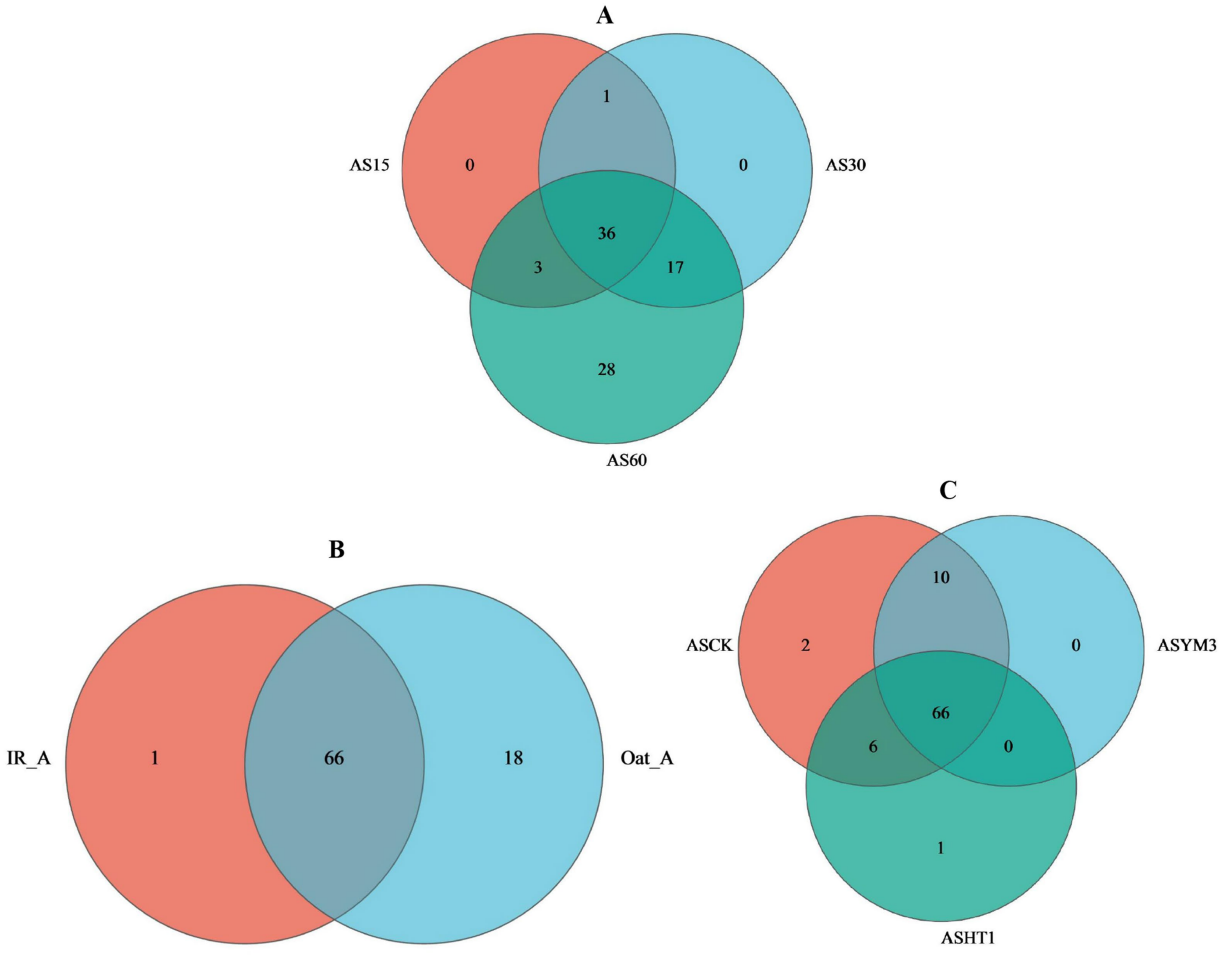
Venn diagram of bacterial communities in silage different ensiling period **(A)**, crop types **(B)**, and additives **(C)**.

**Figure 2:**
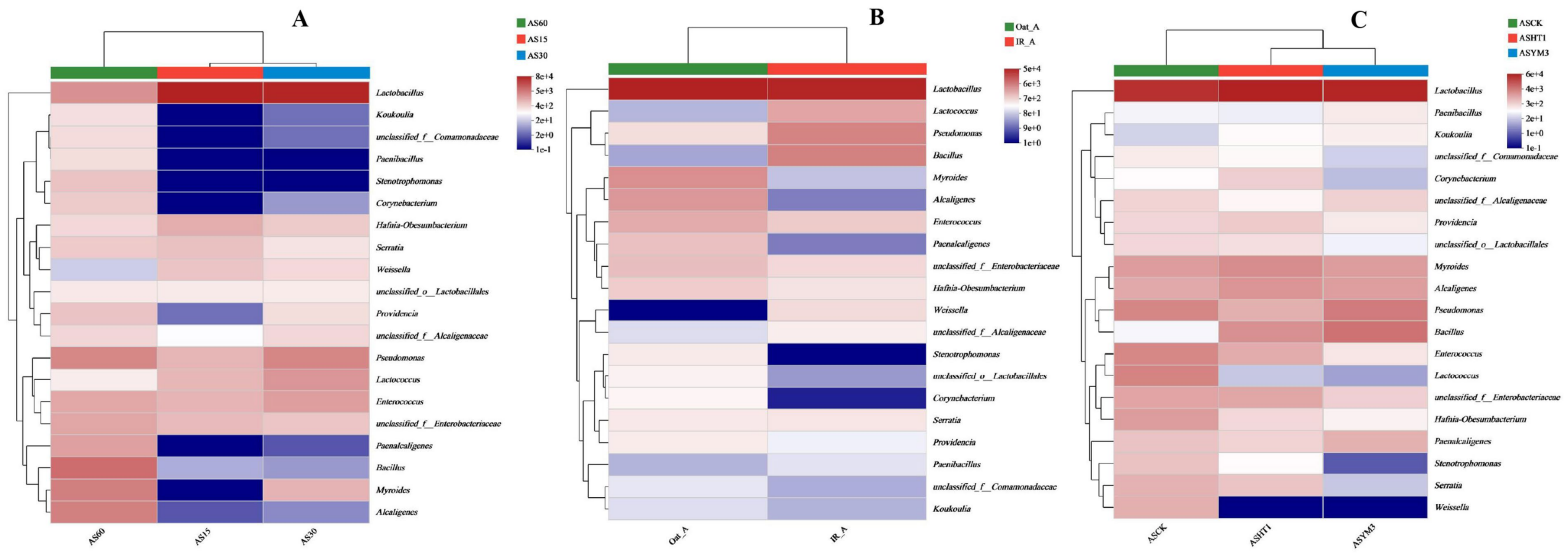
Community heatmap of silage bacterial genus under different ensiling period **(A)**, crop types **(B)**, and additives **(C)**.

During ensiling, AS15, AS30, and AS60 had 4, 12, and 39 OTUs, respectively, yet they shared 56 OTUs in common (accounting for 42.1% of the total) (Figure 1A). Regarding crop type, the IR_A and Oat_A treatments shared 66 OTUs, however, it is noteworthy that the Oat_A treatment had an additional 17 unique OTUs compared to the IR_A treatment (Figure 1B). In total, 84, 76, and 73 OTUs were identified in the silage samples obtained from the ASCK, ASYM3, and ASHT1 treatments, respectively. This study found that the number of OTUs in the ASCK treatment was 11 (nine more than those in the ASHT1 and ASYM3 treatments), although these three treatments also shared 66 OTUs (Figure 1C). The highest relative abundance of *Bacillus* was observed in the AS60 treatment. However, the highest relative abundance of *Lactobacillus* was observed in the AS15 and AS30 treatments (Figure 2A). The community relative abundance of *Lactobacillus* was high and was not affected by crop type or additives (Figures 2B,C).

**Figure 3:**
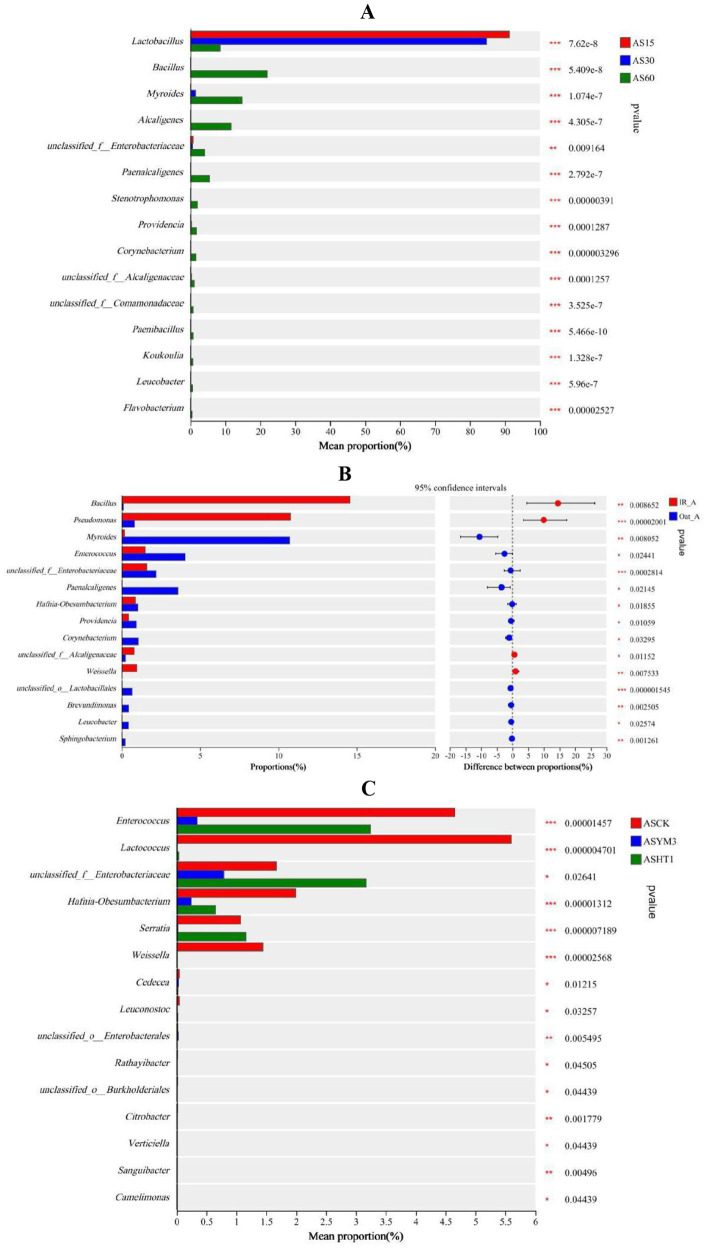
Differences of silage bacterial genus under different ensiling period **(A)**, crop types **(B)**, and additives **(C)**.

At different ensiling durations, the relative abundance of *Lactobacillus* in the AS15 and AS30 treatments was significantly higher than that in the AS60 treatment (*p* < 0.05). In contrast, the relative abundances of various bacteria, including *Bacillus*, *Myroides*, *Alcaligenes*, *Paenalcaligenes*, *Stenotrophomonas*, *Providencia*, and *Corynebacterium* in the AS60 treatment were all higher than in the AS15 and AS30 treatments (*p* < 0.05) (Figure 3A). Among the relative abundances of various bacteria, the relative abundances of *Bacillus*, *Pseudomonas*, and *Weissella* in the IR_A treatment were significantly higher than those in the Oat_A treatment (*p* < 0.05). In contrast, the relative abundances of *Myroides*, *Enterococcus*, *Paenalcaligenes*, *Hafnia-Obesumbacterium*, *Providencia*, *Corynebacterium*, *Brevundimonas*, *Leucobacter*, and *Sphingobacterium* were significantly lower in the IR_A treatment than in the Oat_A treatment (*p* < 0.05) (Figure 3B). The additive significantly affected the relative abundances of bacteria, such as *Enterococcus*, *Lactococcus*, *Hafnia-Obesumbacterium*, and *Weissella* (*p* < 0.05). Compared to the ASHT1 and ASYM3 treatments, the relative abundance of these bacteria in the ASCK treatment was the highest (Figure 3C).

### The interrelationship between organic acids and bacterial communities

**Figure 4:**
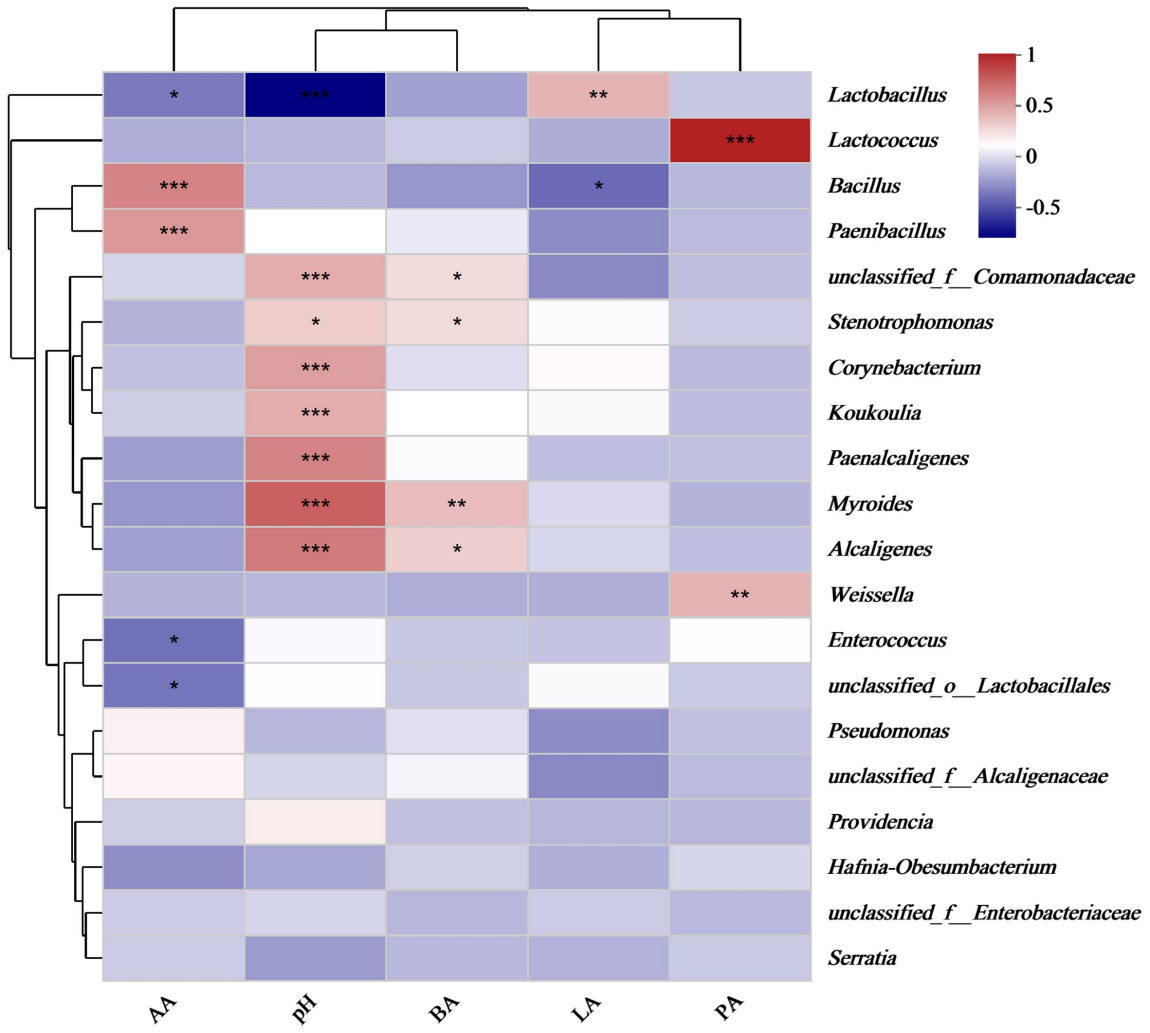
Correlation analysis between organic acids, pH, and bacterial relative abundance. *indicate significant differences at *p < 0.05, **p < 0.01 and ***p < 0.001 respectively.

**Figure 5:**
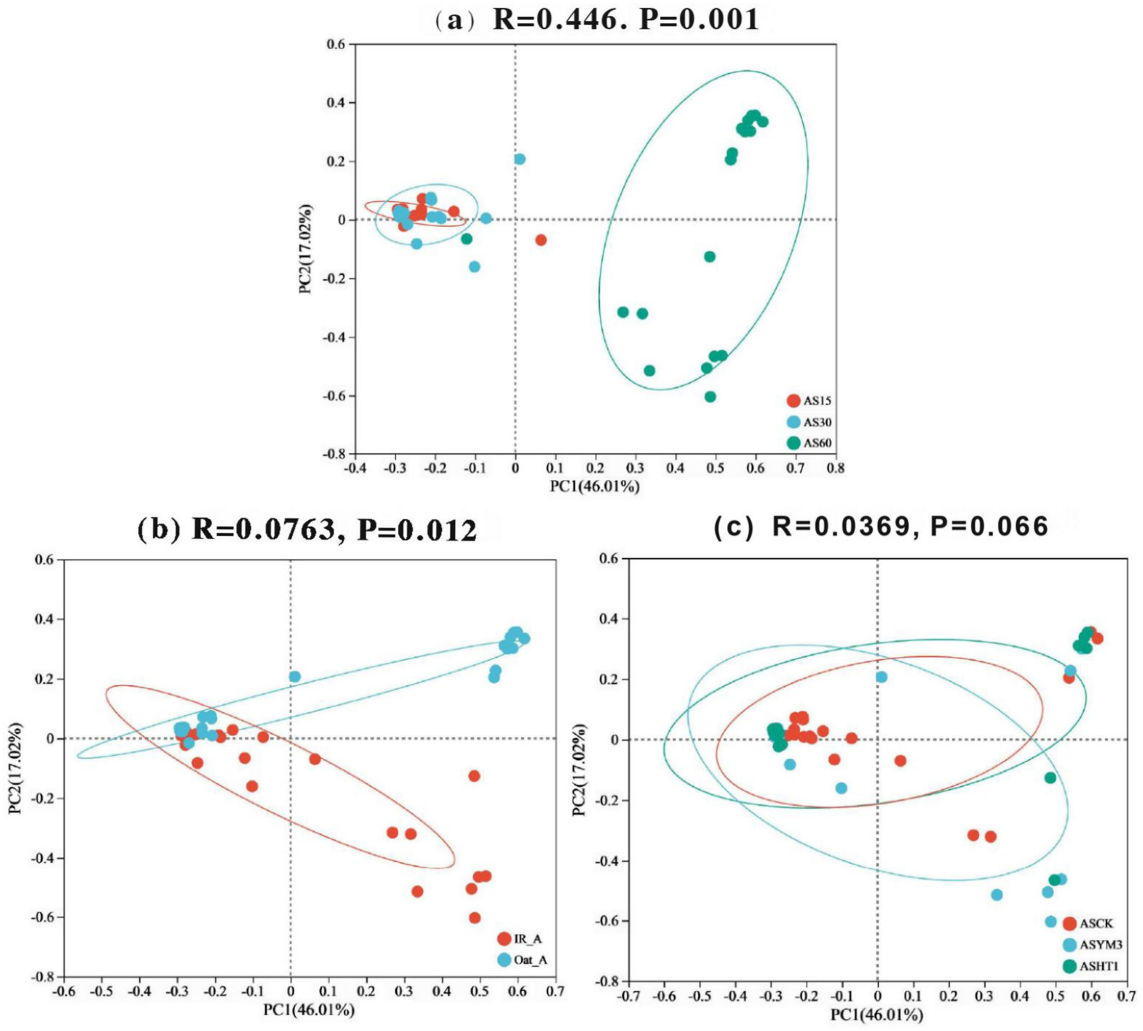
Principal component analysis diagram of bacteria in silage of different ensiling period **(a)**, crop types **(b)**, and additives **(c)**.

After aerobic exposure of the silage, the acetic acid concentration exhibited a significantly positive correlation with the abundances of *Bacillus* and *Paenibacillus* (*p* < 0.05), whereas it was negatively correlated with the abundances of *Lactobacillus* and *Enterococcus* (*p* < 0.05), as illustrated in Figure 4. Additionally, pH was positively correlated with the abundances of *Stenotrophomonas*, *Corynebacterium*, *Koukoulia*, *Paenalcaligenes*, *Myroides*, and *Alcaligenes* (*p* < 0.05). Furthermore, lactic acid concentration was positively correlated with *Lactobacillus* abundance (*p* < 0.05) but negatively correlated with *Bacillus* abundance (*p* < 0.05). Finally, the butyric acid concentration was positively correlated with the abundances of *Stenotrophomonas*, *Myroides*, and *Alcaligenes* (*p* < 0.05). In the principal component analysis, the first and second principal components exhibited variance contribution rates of 46.01 and 17.02%, respectively (Figure 5). Specifically, the 95% confidence intervals of the AS15 and AS30 treatments showed a high degree of overlap but did not intersect with the confidence interval of the AS60 treatment (*p* = 0.001) (Figure 5a). In contrast, in the two crops, the 95% confidence intervals of the IR_A and Oat_A silages showed only minimal overlap (*p* = 0.012) (Figure 5b). However, in the additive treatments, the 95% confidence intervals of the ASCK, ASHT1, and ASYM3 treatments exhibited an extensive overlap (*p* = 0.066) (Figure 5c).

**Figure 6:**
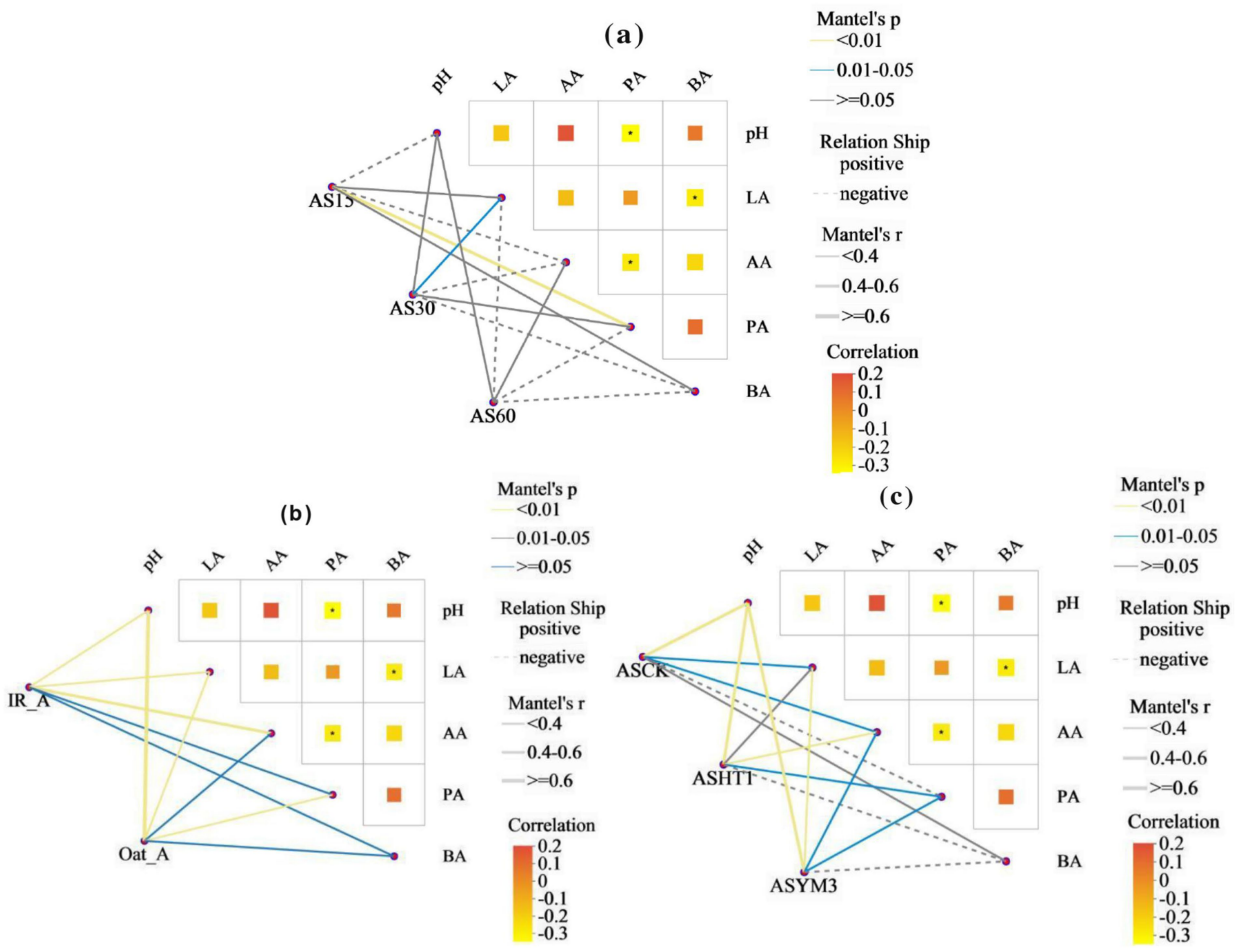
Mantel test heatmap of bacteria in silage of different ensiling period **(a)**, crop types **(b)**, and additives **(c)**.

**Figure 7:**
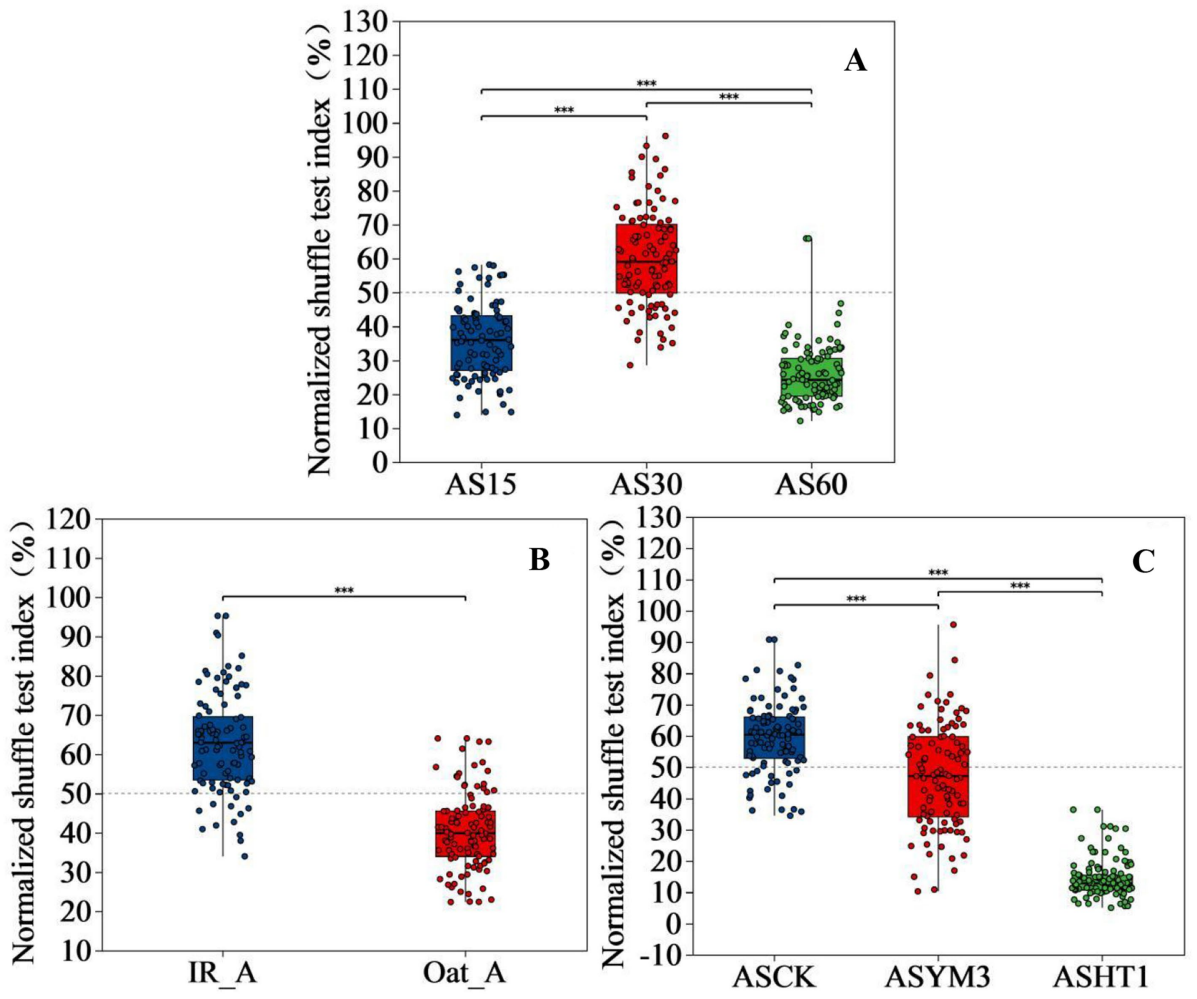
The effect of pesticide concentration on normalized shuffle test index of bacterial communities in silage of different ensiling period **(A)**, crop types **(B)**, and additives **(C)**. *indicate significant differences at *p < 0.05, **p < 0.01 and ***p < 0.001 respectively.

Mantel test analysis revealed that the effects of the ensiling period, crop type, and additives on organic acids and pH values exhibited diverse characteristics (Figure 6). However, a significantly negative correlation between propionic and acetic acid concentrations could not be ignored, and a negative correlation was observed between lactic and butyric acid concentrations (*p* < 0.05). Linear regression analysis of bacterial beta diversity showed that the lactic and acetic acid concentrations, as well as pH, had significant effects on bacterial beta diversity (*p* < 0.05) (Table 2). The ensiling time, crop type, and additive treatments had significant effects on the normalized shuffle test (NST) index (Figure 7).

**Table 2:** Effects of organic acids and pH on the relative abundance of bacteria (*n* = 54).

Items	Linear regression	R^2^	*p* value
pH	y = −26555.737x + 118679.558	0.467	0.000
Lactic acid	y = 627.805x − 46173.234	0.1881	0.0011
Acetic acid	y = −3425.674x + 14278.718	0.0857	0.0317
Propionic acid	y = −384.875x + 396.563	0.0011	0.8121
Butyric acid	y = −1317.390x + 5927.280	0.0249	0.2549

### Effects of ensiling time, crop type and additives on unfavorable silage bacteria after aerobic exposure

Among the top 50 bacterial genera, this study identified 10 undesirable species that were closely related to protein degradation in silage, and detrimental to animal and human health (Table 3). For most undesirable bacterial species, such as *Stenotrophomonas*, *Providencia*, *Paenalcaligenes*, *Myroides*, and *Alcaligenes*, the relative abundances in the AS60 treatment were generally higher than those in the AS30 and AS15 treatments. Among these harmful bacteria, their relative abundances in the Oat_A treatment were higher than those in the IR_A treatment. However, it is noteworthy that in the IR_A treatment, the relative abundances of *Bacillus* and *Pseudomonas* were significantly higher than those in the Oat_A treatment (*p* < 0.05). For *Stenotrophomonas* and *Providencia*, the relative abundances in the ASCK treatment were higher than those in the ASHT1 and ASYM3 treatments. *Pseudomonas* and *Rathayibacter* exhibited relatively consistent trends in the additive treatments, with the relative abundances in the ASYM3 treatment being higher than those in the ASCK and ASHT1 treatments. In summary, extending treatment time typically led to an increase in the relative abundance of the most harmful bacteria. In the crop-type treatments, the Oat_A treatment tended to be more conducive to the growth and reproduction of harmful bacteria. The addition of LAB helped to inhibit the increase in the relative abundance of harmful bacteria.

**Table 3:** The presence of unfavorable bacteria in silage and their specific manifestations in this study.

Species name	Function	Treatment in this study		
		Ensiling time	Crop type	Additives
*Stenotrophomonas*	Can degrade protein (Zi et al., 2021).	AS60>AS30>AS15 (*p* < 0.05)	Oat_A>IR_A (*p* < 0.05)	ASCK>ASHT1>ASYM3 (*p* > 0.05)
*Providencia*	Associated with some diseases in animals (Gupta et al., 2012).	AS60>AS30>AS15 (*p* < 0.05)	Oat_A>IR_A (*p* < 0.05)	ASHT1>ASCK>ASYM3 (*p* > 0.05)
*Pseudomonas*	Production biogenic amine (Li et al., 2024).	AS60>AS30>AS15 (*p* > 0.05)	IR_A>Oat_A (*P* < 0.001)	ASYM3>ASCK>ASHT1 (*P* > 0.05)
*Bacillus*	Protein-degrading bacteria (Hao et al., 2020).	AS60>AS15>AS30 (*p* < 0.05)	IR_A>Oat_A (*P* < 0.05)	ASYM3>ASHT1>ASCK (*p* > 0.05)
*Enterococcus*	Protein-degrading bacteria (Hao et al., 2020).	AS30>AS60>AS15 (*p* > 0.05)	Oat_A>IR_A (*p* > 0.05)	ASCK>ASHT1>ASYM3 (*p* > 0.05)
*Paenalcaligenes*	Implicated in human infections (Wongsurawat et al., 2023).	AS60>AS30>AS15 (*p* < 0.05)	Oat_A>IR_A (*p* < 0.05)	ASYM3>ASCK>ASHT1 (*p* > 0.05)
*Myroides*	Causing aerobic spoilage (Liu et al., 2013).	AS60>AS30>AS15 (*p* < 0.05)	Oat_A>IR_A (*p* < 0.05)	ASHT1>ASYM3>ASCK (*p* > 0.05)
*Alcaligenes*	Degrades urea and releases ammonia, thus increasing pH (Gallo et al., 2021).	AS60>AS30>AS15 (*p* < 0.05)	Oat_A>IR_A (*p* > 0.05)	ASHT1>ASYM3>ASCK (*p* > 0.05)
*Rathayibacter*	Produce extracellular polysaccharides in infected plants resulting in bacteriosis diseases (Murray et al., 2017).	AS60>AS30>AS15 (*p* > 0.05)	Oat_A>IR_A (*p* > 0.05)	ASCK>ASHT1>ASYM3 (*P* < 0.05)
*Citrobacter*	Main contributors to NH_3_ synthesis (Li et al., 2022).	AS60>AS30>AS15 (*p* > 0.05)	Oat_A>IR_A (*p* > 0.05)	ASCK>ASHT1>ASYM3 (*P* < 0.05)

## Discussion

Storing forage infected with leaf spot disease through ensiling not only helps prevent further spread of the disease but also converts forage that may otherwise be discarded due to the disease into valuable feed resources, thereby improving production efficiency and reducing feed costs (Juskiw et al., 2021). Although the silage process aims to create an anaerobic environment to promote LAB fermentation during the actual production, feeding, storage, and transportation processes, silage often encounters the problem of aerobic exposure (Avila and Carvalho, 2020). The primary causes of aerobic exposure included, but were not limited to, improper operations during production, poor feeding and storage management, and vibrations and bumps during transportation. Aerobic exposure increased the oxygen content in silage, thereby promoting the growth and reproduction of aerobic microorganisms such as yeasts and molds (Weiß et al., 2022). These aerobic microorganisms can decompose nutrients, such as lactic acid and the remaining soluble sugar substrates, leading to a continuous rise in pH and temperature (Zhao et al., 2024), ultimately resulting in silage spoilage and deterioration. In this study, regardless of the ensiling period, the difference in crop species, or the variation in additive treatments, even after 7 days of aerobic exposure, the detected LAB counts and their lactic acid production remained higher than the values reported in some published studies (Zhang et al., 2019; Zhang et al., 2023). Simultaneously, the yeast and mold counts, as well as the butyric acid content, remained elevated. These findings suggest that the fermentation quality of silage with leaf spot disease may be affected by complex microbial activity under prolonged aerobic exposure.

After 7 days of aerobic exposure, the LAB count in all silage remained > 7 lg cfu g^−1^ FM, and further extension of the ensiling period triggered an upward trend of yeast and mold numbers under aerobic exposure. The pH of the silage also increased, which can be attributed to the organic acid metabolic pathway of yeast initiated by silage oxygenation. This pathway effectively reduces the lactic acid concentration and promotes an increase in pH (Chen et al., 2022). The significant decrease in lactic acid content as a direct consequence of extended ensiling duration and aerobic exposure further supports this observation. This phenomenon was verified in previous studies (Kung et al., 2018). During silage fermentation, the microbial community structure undergoes significant changes, with certain microorganisms gradually gaining dominance while others are suppressed (Jatkauskas et al., 2024). This evolution of the community structure had a profound impact on the microbial activity characteristics of silage after aerobic exposure. This study found that in silage fermented for 60 days, *Lactobacillus* became the dominant species (data not shown) (Xin et al., 2022), however, their dominance rapidly weakened after aerobic exposure. Furthermore, the relative abundances of harmful bacteria, represented by *Myroides* and *Alealigenes*, increased sharply, significantly increasing the safety risk of the silage. In contrast, in silage from the AS15 and AS30 treatments, even when exposed to oxygen, the relative abundance of *Lactobacillus* remained high and dominant. Particularly in the AS15 treatment, the relative abundances of harmful bacteria were much lower than those in the AS60 treatment, indicating a healthier bacterial community structure. This finding suggests that in silage infected with leaf spot disease, shortening the ensiling period helps optimize the silage bacterial community structure after aerobic exposure, reducing the growth potential of harmful bacteria, thereby enhancing the safety and quality of feed. Therefore, reasonable regulation of the ensiling period is the key to improving the bacterial community structure and ensuring silage safety.

This study observed that compared to the IR_A treatment, the Oat_A treatment exhibited higher acetic acid and lower butyric acid concentrations (*p* < 0.05). Notably, the relative abundances of *Bacillus* and *Pseudomonas* in the IR_A treatment were higher than those in the Oat_A treatment, and both bacterial groups showed a significantly positive correlation with the acetic acid concentration. Conversely, the relative abundances of *Myroides*, *Alcaligenes*, and *Stenotrophomonas* in the Oat_A treatment were higher than those in the IR_A treatment, and all demonstrated a significantly positive correlation with the butyric acid concentration. This suggests that different bacteria have distinct mechanisms for the production and regulation of acetic and butyric acids (Kung et al., 2018). Specifically, *Bacillus* and *Pseudomonas* may have promoted the production of acetic acid or inhibited the generation of butyric acid (Mumtaz et al., 2019; Chen Y. et al., 2021), leading to higher acetic acid content in the IR_A treatment, whereas *Myroides*, *Alcaligenes*, and *Stenotrophomonas* may possess the ability to enhance butyric acid production or inhibit acetic acid formation, resulting in higher butyric acid content in the Oat_A treatment. In the current study, the relative abundances of *Lactobacillus*, *Lactococcus*, *Pseudomonas*, and *Bacillus* in the IR_A treatment were dominant, far exceeding those of other species. Compared to the Oat_A treatment, the number of OTUs exhibited a noticeably decreasing trend. However, the NST index of the IR_A treatment was higher than that of the Oat_A treatment (*p* < 0.001). This phenomenon indicates that despite the relatively concentrated microbial species in the IR_A treatment, microbial system activity was exceptionally vigorous, potentially owing to the enhanced microbial metabolic activity prompted by the high abundances of specific species. Additionally, the higher NST indices revealed that the environmental stability of the IR_A samples was relatively poor, which may have been triggered by microbial interactions or changes in environmental factors. Consequently, we speculated that the characteristics of this microbial community structure may have profoundly influenced certain physiological or biochemical properties of the IR_A treatment, further affecting its overall performance and behavior. Unfortunately, this study did not conduct a more in-depth analysis of silage metabolites, thus failing to verify this speculation.

In adverse environments such as high pH, temperature, and osmotic pressure, *Enterococcus* typically demonstrated stronger survival, stress resistance, and proliferation capabilities compared to other LAB (Acciarri et al., 2023). Furthermore, during the initial stages of silage fermentation, *Enterococcus faecalis*, a primary lactic acid bacterium, rapidly produces lactic acid, albeit with relatively weak acid tolerance (Wang et al., 2020). Subsequently, as fermentation progressed, *Enterococcus* was gradually replaced by the more acid-tolerant *Lactobacillus* (Parvin et al., 2010). However, when exposed to aerobic conditions, despite its lack of acid tolerance, *Enterococcus faecalis*’s strong oxygen tolerance allowed it to survive. The results of this study confirm this characteristic of *Enterococcus*, with higher pH values and lower lactic acid concentrations in the ASCK treatment. Even under aerobic conditions, the effect of LAB additives remained significant. For example, compared to the ASCK treatment, the ASHT1 and ASYM3 treatments effectively inhibited the number of OTUs by increasing the content of lactic and acetic acids, while optimizing microbial competition and environmental stability. Additionally, this study found that the types of bacteria closely related to human health and nutritional loss were generally higher in the ASCK treatment than in the ASHT1 and ASYM3 treatments, indicating that LAB additives effectively improved the health and nutritional value of silage exposed to air. This was independent of the health status of the plant.

Notably, based on the analysis of the NST index, only the additive treatment affected the stability of the silage environment by adjusting the number of OTUs, whereas silage duration and crop type were more likely to influence the stability of the silage environment by regulating the types and quantities of metabolites. For example, the content of l-phenylalanyl-l-proline increases with the number of *Enterococcus*, whereas the content of glutamylvaline decreases as the number of *Enterococci* increases (Fu et al., 2022). *Bacillus* species can grow anaerobically either by using nitrate or nitrite as terminal electron acceptors or through fermentation (Ramos et al., 2000) and are metabolized to produce acetic, formic, and succinic acids (Ouhib-Jacobs et al., 2009). The influence of pH and lactic acid concentration on the relative abundance of bacteria was limited, further demonstrating that changes in acid-tolerant microorganisms are crucial for the silage environment. Microorganisms that are capable of producing acetic, propionic, and butyric acids are of secondary importance.

## Conclusion

Forage affected by leaf spot disease can still be used for silage, even when exposed to air for up to 7 days. However, as the ensiling period progressed, the fermentation quality and health of the silage exposed to air gradually decreased. In comparison, the silage fermentation quality and health benefits of Italian ryegrass under aerobic conditions were superior to those of oats. The use of LAB additives can enhance the fermentation quality and health of aerobically exposed silage. Therefore, when silage has to be exposed to air, this study recommends that Italian ryegrass be inoculated with *Lactobacillus rhamnosus* HT1 and used within 15 days of ensiling.

## Data Availability

This data is available through the National Microbiology Data Center (NMDCR1693659043309: Index of /NMDCSra/public/202309/NMDC40041972/NMDCE1693659043305/NMDCR169365904 3309/).
